# Capturing Genomic Evolution of Lung Cancers through Liquid Biopsy for Circulating Tumor DNA

**DOI:** 10.1155/2017/4517834

**Published:** 2017-03-14

**Authors:** Michael Offin, Jacob J. Chabon, Pedram Razavi, James M. Isbell, Charles M. Rudin, Maximilian Diehn, Bob T. Li

**Affiliations:** ^1^Thoracic Oncology Service, Division of Solid Tumor Oncology, Department of Medicine, Memorial Sloan Kettering Cancer Center, Weill Cornell Medical College, New York, NY, USA; ^2^Institute for Stem Cell Biology and Regenerative Medicine, Stanford University, Stanford, CA 94305, USA; ^3^Stanford Cancer Institute, Stanford University, Stanford, CA 94305, USA; ^4^Department of Radiation Oncology, Stanford University, Stanford, CA 94305, USA; ^5^Department of Medicine, Memorial Sloan Kettering Cancer Center, Weill Cornell Medical College, New York, NY, USA; ^6^Thoracic Service, Department of Surgery, Memorial Sloan Kettering Cancer Center, Weill Cornell Medical College, New York, NY, USA

## Abstract

Genetic sequencing of malignancies has become increasingly important to uncover therapeutic targets and capture the tumor's dynamic changes to drug sensitivity and resistance through genomic evolution. In lung cancers, the current standard of tissue biopsy at the time of diagnosis and progression is not always feasible or practical and may underestimate intratumoral heterogeneity. Technological advances in genetic sequencing have enabled the use of circulating tumor DNA (ctDNA) analysis to obtain information on both targetable mutations and capturing real-time Darwinian evolution of tumor clones and drug resistance mechanisms under selective therapeutic pressure. The ability to analyze ctDNA from plasma, CSF, or urine enables a comprehensive view of cancers as systemic diseases and captures intratumoral heterogeneity. Here, we describe these recent advances in the setting of lung cancers and advocate for further research and the incorporation of ctDNA analysis in clinical trials of targeted therapies. By capturing genomic evolution in a noninvasive manner, liquid biopsy for ctDNA analysis could accelerate therapeutic discovery and deliver the next leap forward in precision medicine for patients with lung cancers and other solid tumors.

In the current era of precision medicine, molecularly targeted therapies against oncogene driven lung cancers have been shown to achieve initial response rates of up to 70% [1]. Unfortunately, these medications inevitably fail over time due to the emergence of acquired resistance [2, 3]. These resistance mechanisms may emerge and persist through Darwinian evolution of tumor clones under therapeutic pressure [4]. The selective pressure favoring drug resistant malignant clones during treatment may alter the molecular profiles of tumors and their associated drug sensitivities, necessitating repeat biopsies to help guide further therapy. However, repeat biopsies are often impractical for patients and may fail to adequately reflect intratumoral heterogeneity, both of which represent substantial impediments to clinical care and therapeutic advances [5]. Recent technological advances in sequencing of circulating tumor DNA (ctDNA) have enabled the identification of tumor derived somatic alterations from plasma, urine, and cerebrospinal fluid (CSF) with high degrees of sensitivity and specificity [6, 7]. These advances comprise a unique opportunity to uncover novel mechanisms of acquired resistance to targeted therapies and capture genomic evolution in patients with molecular subsets of lung cancers in a noninvasive and universally obtainable way, the “liquid biopsy” (Figure 1).

Liquid biopsies can be obtained with relative ease from plasma (blood volume needed: 10–20 mL) [8], lumbar puncture (CSF needed: 1-2 mL) [6, 9], or urine (urinary volume needed: 30–50 mL) [10]. Once the liquid biopsy is obtained, the cell-free DNA (cfDNA) can be extracted and analyzed for tumor-specific alterations using any of a variety of techniques. Digital polymerase chain reaction (PCR) and next generation sequencing (NGS) are the primary methods of ctDNA analysis. Two of the most common digital-PCR based methods are Droplet Digital PCR (ddPCR) and BEAMing (beads, emulsions, amplification, and magnetics) [11, 12]. Both of these methods leverage emulsion PCR, in which droplets containing individual DNA fragments are generated, allowing for DNA molecules to be amplified independently of one another. Sequences differentiating fluorescently labeled probes are then used to distinguish droplets containing mutant or wild-type alleles of interest. Counting of individual droplets enables more precise quantitation of mutant allele fractions than traditional reverse transcriptase-PCR (RT-PCR) based approaches [13]. In contrast, hybrid capture NGS selects the portions of the genome containing reference oncogenic mutations prior to sequencing to enrich the yield of the assay. Briefly, whole genome DNA libraries are generated from cell-free DNA via ligation of adapters and PCR. The genomic regions of interest are then selected out of the amplified libraries by hybridization capture using oligonucleotides or “baits” complementary to these regions, for subsequent enrichment. After another round of PCR, the enriched libraries are sequenced [14]. Another method, amplicon-based NGS, utilizes a multiplexed PCR to create a pool of amplified oligo-set primers which can be used to label a variety of target regions for “hotspots” of recurrent somatic mutations [15].

Each analysis method has its own diagnostic niche. Digital PCR is rapid, allows for quantitation of mutant alleles at very low concentrations, and is relatively inexpensive. However, it requires a priori knowledge of the specific mutations of interest and cannot be used to detect rearrangements unless the exact genomic breakpoint is known, and multiplex analysis of more than a few mutations is challenging [16]. Hybrid capture-based NGS allows multiplex analysis of thousands of genomic positions and in addition to single nucleotide variants and short insertions/deletions can readily detect rearrangements and copy number variation. Recent advances in NGS technology have made it possible to achieve similar or even better analytical sensitivity for ctDNA detection as digital PCR through molecular barcoding and digital error suppression [16, 17]. However, NGS-based methods are relatively more expensive than digital PCR and have longer turnaround times.

Historically, analysis of a tissue biopsy has been the primary method used to study acquired resistance in lung cancer patients treated with targeted therapies [18–20]. However, the need to attain a repeat biopsy following progression is often a significant obstacle to performing such analyses. Recent studies have demonstrated that plasma ctDNA may be used for the early recognition and definition of mechanisms of acquired resistance to targeted agents in oncogene driven lung cancers [21, 22]. This includes detecting the emergence of* EGFR* T790M in plasma of patients with* EGFR* mutant lung cancers treated with erlotinib, which accounts for the majority of treatment failures [13]. The importance of studying acquired resistance mechanisms is further highlighted by the recent development of third-generation mutant-selective EGFR inhibitors used to overcome* EGFR* T790M, including osimertinib which has recently gained FDA approval and has been adopted as a standard of care [23]. In this setting, detection of T790M in plasma was shown to be just as predictive of osimertinib response as detection of T790M in tissue [2]. Additionally, novel resistance mechanisms to osimertinib and rociletinib such as* EGFR* C797S and L798I mutations, as well as previously described mechanisms such as* MET* amplification, and activating mutations in* PIK3CA* have been identified and/or characterized by sequencing of plasma ctDNA (Figure 2) [24, 25]. It is worth pointing out that, with longitudinal serial use of ultrasensitive plasma NGS assays, subclonal mutations may be detected before they become clinically relevant. In the recently reported AURA3 trial, plasma* EGFR* T790M status independently predicted improved outcomes for patients with* EGFR* mutant lung cancers who received osimertinib versus platinum/pemetrexed chemotherapy [26, 27]. However, it is still unclear whether there is benefit in initiating new targeted treatment early upon detection of plasma T790M at subclonal status when tissue T790M may well be negative, compared to the current standard of care of switching treatment only upon clinical progression of disease. Prospective clinical trials will be needed to answer this question.

In a study by Murtaza et al., the ability for liquid biopsy to obtain the same mutational information as traditional biopsy was explored in the setting of patients with metastatic lung, ovarian, and breast cancers. In this study, serial plasma ctDNA samples were evaluated over the course of 1-2 years and demonstrated increased mutant allele fraction at the time of therapy resistance [28]. This helped to establish proof of concept that exome wide analysis of ctDNA can complement traditional biopsies at disease progression to capture clonal evolution. Subsequently, similar results were achieved by the more cost-effective approach of targeted NGS [17, 27]. No invasive procedure is without the possibility of morbidity; the adverse event rate including pneumothorax reported from thoracic biopsies, for example, may be up to 19% [29–33]. As ctDNA becomes more widely available and the methodologies further refined, the ability to utilize this noninvasive method to obviate the need for repeat tissue biopsy at the time of progression is becoming a reality in the clinic [34, 35].

Beyond simply reflecting the information obtained by invasive tissue biopsies, ctDNA analysis may offer a more comprehensive and integrated view of systemic evolution of cancer across multiple sites. Discordance of mutational status in primary and metastatic lesions has been shown to be as high as 28% and 24% in* EGFR* and* KRAS*, respectively, in a cohort of 25 patients with stage IV non-small-cell lung cancers (NSCLC) [36]. Additionally, using CAPP-Seq based ctDNA analysis, Chabon et al. recently found evidence for multiple resistance mechanisms in 46% of lung cancer patients following treatment with first-line EGFR TKIs [25], while prior tissue biopsy based studies have reported heterogeneity of resistance mechanisms in only 5%–15% of patients [20–22, 25]. CtDNA is uniquely able to address spatial heterogeneity and the evolution of a systemic disease by sampling the systemic circulation. In this way, ctDNA is poised to be the next major tool in our diagnostic arsenal to help refine systemic treatment decisions for patients with lung cancers.

In addition to defining driver mutational status in the metastatic setting, ctDNA analysis can also be applied to analysis of differences in tumor composition between the central nervous system (CNS) and the periphery, a phenomenon well characterized recently by Brastianos et al. [37]. In a study by Pentsova et al., ctDNA from CSF samples were used to show that the CNS compartment harbors clinically relevant genomic alterations which show promise for the monitoring of both primary CNS tumors and metastatic lesions [6]. Genotyping the primary tumor alone can miss substantial targeted therapy opportunities for CNS metastases; however, routine brain biopsies are simply unfeasible. CtDNA analysis of cerebrospinal fluid (CSF) is emerging as a novel approach to address this unmet need through a far less invasive procedure, lumbar puncture, performed at the bedside or the neurooncologist's office.

Several studies suggest that malignancy involving the CNS, be it a primary CNS tumor or a metastatic lesion, poses a unique challenge for acquisition of ctDNA representative of the central lesion in the peripheral blood as shed material may not predictably cross the blood-brain barrier (BBB) [38, 39]. De Mattos-Arruda et al. showed that ctDNA was more abundant in the CSF and more representative of CNS tumor genetic mutational status than ctDNA from plasma in the same individual [9, 39]. There is also data demonstrating differences in resistance mechanisms to driver targeted therapy in the CNS versus periphery, thought to be attributable to reduced BBB penetration of the drug. This lower concentration of medication in the CSF allows for differences in selective pressure, leading to alternative mechanisms of resistance in the CNS compartment [40, 41]. In a cohort of 12 patients with progressive CNS disease burden on targeted therapy with TKIs (EGFR, ALK, HER2, or BRAF), 4 were found to have mutations on CSF ctDNA which conferred resistance to targeted treatment that was not otherwise seen in the periphery [19]. Parallel analyses of plasma and CSF pools can provide a unique and comprehensive view of tumor evolution in vivo. Determining the ideal time for CSF ctDNA testing is still under investigation in patients with lung cancers and CNS metastases. A concept is to consider CSF ctDNA analysis compared to plasma ctDNA when a patient has clinical progression in the CNS compartment discordant from the periphery to look for distinct mutational profile, as brain biopsy is most often not feasible in this setting. With the clinical development of CSF ctDNA, we can discover the genomic profile and evolution of CNS metastases without performing invasive brain biopsies and tailor CNS targeted treatments toward their specific genetic aberrations.

Given the noninvasive nature of liquid biopsy and the rapidly improving methodologies for detecting ctDNA, the ability for real-time minimally invasive monitoring of the emergence of resistance mechanisms is quickly becoming a reality. Therefore, we advocate for the incorporation of ctDNA analysis in clinical trials of targeted therapies for the systematic interrogation of genomic evolution of lung cancers. Continued clinical research is vital as we incorporate ctDNA into everyday clinical practice. Researches in optimization of timing, frequency, and choice of liquid biopsy testing in the clinical setting along with the ongoing technical refinements that are expected to bring down the cost of genetic sequencing from ctDNA are keys to help mitigate the financial toxicity for patients in the future. By capturing genomic evolution, ctDNA analysis could accelerate therapeutic discovery and deliver the next leap forward in precision medicine for patients with lung cancers and other solid tumors.

## Figures and Tables

**Figure 1:**
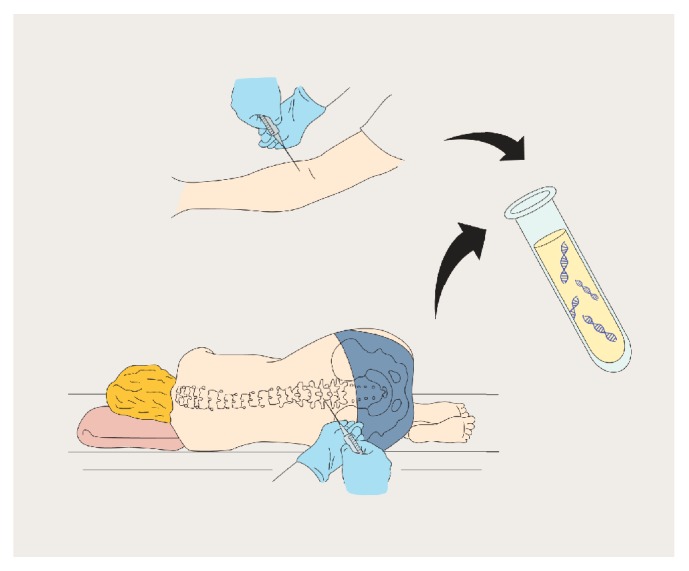
Illustrative representation of the noninvasive and universally obtainable methods of liquid biopsy for ctDNA from plasma and CSF which can be sequenced to ascertain oncogenic drivers and resistance mechanisms.

**Figure 2:**
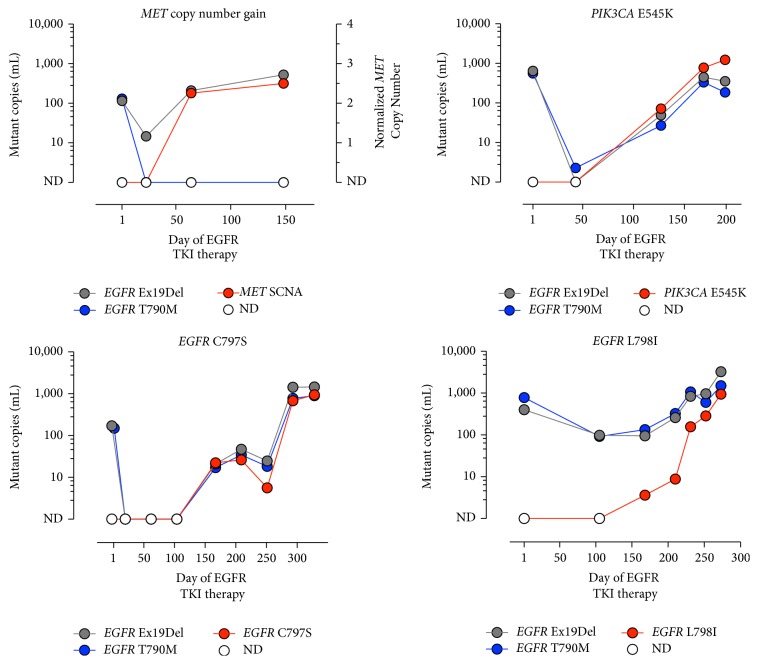
Application of NGS-based ctDNA analysis for identification of mechanisms of acquired resistance in lung cancer patients treated with EGFR targeted therapies. Serial ctDNA measurements from four different patients with T790M mutant tumors treated with the third-generation T790M-selective EGFR tyrosine kinase inhibitor (TKI) rociletinib. Activating mutations in EGFR are shown in grey, the T790M resistance mutation is shown in blue, and emergent resistance alterations are shown in red. Serial ctDNA measurements were performed using the hybrid capture NGS-based Cancer Personalized Profiling by deep Sequencing (CAPP-Seq) approach [20].
